# Supplementary material for: Identification of photoperiod-induced specific miRNAs in the adrenal glands of Sunite sheep (Ovis aries)
Source: Front Vet Sci. 2022 Jul 22;9:888207. doi: 10.3389/fvets.2022.888207 (PMC9354845; doi:10.3389/fvets.2022.888207)
Supplement: Supplementary file 1 [file Data_Sheet_1.ZIP › Supplementary file/miRDeep2 file.zip!/miRDeep2 file/Novel_Structure/SPLP42A2/Novel_335.pdf]

Provisional ID : NC_019484.2_42907
Score total : 1.8
Score for star read(s) : -1.3
Score for read counts : 0
Score for mfe : 1.5
Score for randfold : 1.6
Score for cons. seed :
Total read count : 253
Mature read count : 253
Loop read count : 0
Star read count : 0

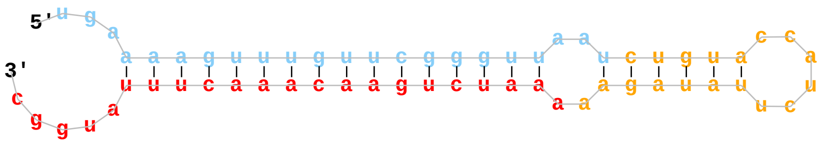

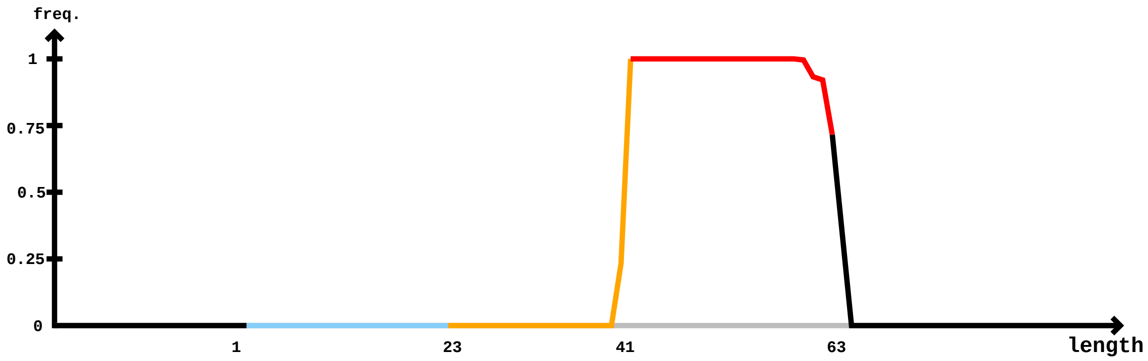

Star Mature

```
5'- acaauccuagguguuggcugaaaaguuuguucggguuaaaucuguaccaucuuauagaaaaaucugaacaaacuuuauggcuaacccaguauuuucucuucuuaccugaa   -3'   exp
    .........((.((((((((.((((((((((((((((..((((((......))))))..))))))))))))))))).))))))))))........................    reads   mm   sample
    ..........................................................aaaaucugaacaaacuuuU.................................    1       1    seq
    ..........................................................aaaaucugaacaaacuuuUu................................    15      1    seq
    ..........................................................aaaaucugaacaaacuuuUugg..............................    39      1    seq
    ..........................................................aaaaucugaacaaacuuuUuggc.............................    4       1    seq
    ...........................................................aaaucugaacaaacuuuUu................................    1       1    seq
    ...........................................................aaaucugaacaaacuuuUug...............................    3       1    seq
    ...........................................................aaaucugaacaaacuuuUugg..............................    13      1    seq
    ...........................................................aaaucugaacaaacuuuUuggc.............................    177     1    seq
```
